# Performance Evaluation of Two Slow-Medium Growing Chicken Strains Maintained under Organic Production System during Different Seasons

**DOI:** 10.3390/ani11041090

**Published:** 2021-04-11

**Authors:** Ainhoa Sarmiento-García, Isabel Revilla, José-Alfonso Abecia, Carlos Palacios

**Affiliations:** 1 Departamento de Construcción y Agronomía, Facultad de Agricultura y Ciencias Ambientales, Universidad de Salamanca, Av. de Filiberto Villalobos 119, 37007 Salamanca, Spain; asarmg00@usal.es; 2 Area de Tecnología de los Alimentos, Escuela Politécnica Superior de Zamora, Universidad de Salamanca, Av. de Requejo 33, 49029 Zamora, Spain; irevilla@usal.es; 3 Instituto Universitario de Investigación en Ciencias Ambientales de Aragón, Facultad de Veterinaria, Universidad de Zaragoza, Calle de Miguel Servet 177, 50013 Zaragoza, Spain; alf@unizar.es

**Keywords:** climate, environment, growth, organic chicken, slow-medium line, weather period

## Abstract

**Simple Summary:**

Expected thermal variations as a consequence of climate change represent a challenge for alternative poultry production because animals graze abroad for long periods of time, where slight variations in ambient temperature may cause negative effects on their productive development. Changes in temperature have been described as factors capable of influencing the development of animals. The objective of this study was to evaluate weather conditions and strain affect the development of slow-medium growing chickens raised in an organic system. No differences were shown between both strains studied; this indicates the high similarity between the strains used. This fact would allow the farmer to include one strain or another in his farm indifferently. Better development was observed in chickens that had been raised under cooler conditions (S1) in organic systems. This fact could be justified by the greater difficulty that chickens find to dissipate heat in warmer environments, which impairs their productive development. In this way, whenever possible, it would be recommended to raise the largest number of chickens during the coldest season.

**Abstract:**

A total of 160 1-day-old medium-growing male chicks (*Gallus gallus domesticus*) were raised for 120 days in a certified organic farming system. A total of two strains were studied (Coloryield, CY; RedBro, RB). Overall, two weather periods were considered based on the outdoor temperature, being S1 colder than S2. In total, 40 chicks per strain were assigned to each period (*n* = 80). Chickens were fed ad libitum with the same organic feeds. In the first month, chickens were kept indoors and, from day 30, they had access to the pasture. Slaughter live weight (LW), average daily gains, (ADG), the feed conversion ratio (FCR), and mortality rates did not differ between the two strains. LW was (*p* < 0.05) higher in the S1 and a trend (*p* = 0.084) was observed for ADG, which was higher in S1. No differences were found for feed intake, FCR, and mortality rates between weather periods. There were no differences for coefficient of variation (CV) between the strains studied, nevertheless, CV for LW in S2 was increased. Differences in the productive performance between these strains raised in organic production systems were slight. However, chickens raised in S1 had a better performance. It would be preferable to raise chickens in these weather conditions whenever possible.

## 1. Introduction

Consumers’ interest in organic and natural poultry products is expanding. The organic market has annually grown by 20% for the past decade [1,2]. Consumers’ interest in healthy foods and animal welfare has promoted the growth of organic production systems, which are supposedly environmentally friendly production methods that keep animals in good health and at high welfare standards [3,4].

Organic farming is a certified production system, it is regulated by European Commission (CE)–no 2018/848, which requires producers to comply to very strict standards in the production, management, and feeding of animals. The EC Regulation 889/2008 states that, in organic production, the choice of poultry breed should take into account the capacity of the breed to adapt to local environmental conditions. However, the use of local breeds is not compulsory, and the standard does not indicate which genotypes should be employed in organic systems [5]. Even this regulation (CE 2018/848) does not allow the use of fast-growing lines; only medium-slow growing hybrids or pure slow growing lines are permitted. In contrast, the US legislation allows raising fast-growing chickens in organic farms. So, there is limited information on trials that evaluate available strains of chickens for organic production in accordance with EU legislation [6]. Most of the studies on these production systems have evaluated fast-growing commercial hybrids vs. pure or slow-growing lines [1,6,7,8].

Native chicken lineages have desirable characteristics in open-field productions, such as resistance to some diseases and hardiness in adverse environmental conditions [1]. Slow-growing chickens have a good ability for grazing, which favors the intake of bioactive substances (vitamins, antioxidants, and fatty acids) contained in the forage [8]. Compared to fast-growing chickens, native chicken and their hybrids show less weight gain and have a smaller proportion of pectoral muscle in the carcass; however, their meat has many qualities that are valued by consumers [1,9] Despite the apparent advantages of including those strains in open-field production systems, the use of pure or native lineages has been questioned because of their low productive performances. For this reason, the use of medium-slow growing hybrids is an alternative for farmers that allows them to overcome the productive deficiencies that pure slow-growing strains can present.

Control of environmental conditions is an important difference between intensive and alternative production chicken farming. Thermal comfort of the animals is essential because high or low temperatures (T) can affect their behavior, performance, and production. The predicted thermal variations caused by climate change pose a challenge for open-field poultry production, where small changes in ambient T have a negative effect on their development (Intergovernmental Panel on Climate Change. 2014). High ambient temperature and relative humidity (RH), particularly in summer, create conditions of almost permanent thermal discomfort for birds, which is a factor that has a significant effect on their production [10]. Numerous meteorological factors can markedly affect the animal’s body heat exchange and, therefore, their energy requirements, which impacts productive performance [11].

The aim of this study was to determine differences in growing rates between two strains of medium-slow growing chickens reared in a certified organic farm by a national administration (ES-ECO-016-CL). Moreover, the effect of weather conditions on the performance of both strains was also evaluated. This study was carried out in an organic farm; therefore, the regulation of this production was complied.

## 2. Material and Methods

### 2.1. Ethics Statement

This study was carried out with farm animals, which due to their characteristics did not require a special certification according to the method of breeding laboratory animals. Nevertheless, these procedures were authorized by the Animal Experimentation Service (SEA) of the University of Salamanca, in line with the standards set by the Confederation of Scientific Societies of Spain (COSCE) (Project Identification Code IDE2019/041). The chickens were reared in accordance with the minimum standards for the protection of chickens kept for meat production of the European Directive (2007/43/EC).

### 2.2. Animals and Experimental Design

A total of 160 one-day-old male chicks (*Gallus gallus domesticus*) were raised for 120 days in a certified organic farm. Chicks were vaccinated against Newcastle disease and Infectious Bronchitis. A total of two strains were studied (Coloryield, CY, *n* = 80, and RedBro, RB, *n* = 80). Coloryield is an intermediate-slow-growing hybrid named NewCY57, which comes from a cross between the male New Coloryield and the female JA57Ki (Hubbard^®^). Redbro an intermediate-slow-growing hybrid named REDJA Ki, which comes from a cross between the male RedBro and the female JA57Ki (Hubbard^®^). The chicks used in the study were two slow-medium growing chickens’ line specialized in meat production. The chicks come from a Spanish conventional farm. However, they are certified as organic production because they are raised under organic guidelines. In addition, the chickens remain at the destination farm for a period of more than 70 days, which allows them to be sold as organic chickens.

In total, 40 animals of each strain were assigned to two different weather periods, based on the outdoor temperature. The weather period (S1 and S2) was determined based on the conditions that affected the chickens from the second month of life (which is when the animals had access to the outdoor facilities) until their sacrifice (120 days of age). The weather periods will be described later in Section 2.3. A total of 160 animals were used. A total of 10 replica were carried out. Each replica had 8 chicks per weather period, 4 of each breed. Each replication was separated from the other replications, allowing only visual contact through separation fences.

During the first month of their life, the chickens were kept with a flock density of 35 chickens/m^2^ in clean and disinfected reception houses under controlled environmental conditions, with barley straw as litter following organic rules (EC Regulation 834/2007 and 889/2008). The temperature range was 25 to 35 °C, with a RH of 65–75%. Incandescent lights (30 lx) at bird level provided heat and light.

Once the chickens reached the age of 30 days, the floodgates were opened to allow access to the outdoor areas during daylight hours, which varied with the natural time of the year, without being supplemented with artificial light at any time. Chickens had no contact with other animals in the areas. The dimensions of the outdoor areas were 8m^2^ per chicken. Chickens had access inside the barn throughout the day for both breeding periods to guarantee their comfort. The chickens had access to different vegetables and shrubs that grew in the outdoor areas. All outdoor areas were completely vegetated. Chickens eat barley and crops that the farmer grows in the outdoor areas. Chickens had access to the pasture all year. In winter, chickens have access to plants that grow naturally in the parks like clovers (*Trifolium pratense*) and grasses (mixture of *Poa pratensis, Lolium perenne* and *Festuca rabra*). In addition, the chickens receive dry remains from the vegetable production of the previous season.

The birds were kept in shelters only during the night to protect them from predators.

The chickens were fed ad libitum with the same certified organic diet (starter diet from day 1 to day 30, and finisher diet from day 30 to day 120). Table 1 shows the ingredients and chemical composition of both diets. The diets were provided by a certified organic feed factory (Coslada, Madrid, Spain). This feed fully complies with the requirements for the requirements of broilers [12] and the organic farming legislation. Water was always available.

### 2.3. Weather Period

A total of 80 chickens were used for each weather period (40 per strain). The weather period S1 and S2 were based on the ambient temperature in the outdoor yards, which the birds were exposed to from day 30 until slaughter (day 120). Climatologic values were obtained from the database of the network of agrometeorological stations of the Agroclimatic Information System for Irrigation (SiAR). The registered data correspond to the climatic conditions of Venialbo (Zamora, Spain) which is located in the northwest region of Spain. Venialbo is situated within the longitude −5.54 and 41.39 latitude. The climate is continental, it is characterized by hot-dry summers and wet and cool winters. S1 corresponded to the winter–spring months (November–May), and S2 corresponded to the summer–autumn months (June–October) (Table 2). The mean, maximum, and minimum T, thermal amplitude, and mean and minimum RH differed significantly (*p* < 0.0001) between the two weather periods. Those conditions are common in raising chickens in organic production systems in Spain. The average temperature of period S1 was lower than that recorded in period S2 (9.32 vs. 16.86 °C). While the values of humidity (75.14 vs. 60.59%) and rainfall (1.64 vs. 0.87 mm) were higher in period S1. This is justified by the climatic conditions of the geographical area where the experiment was carried out.

The temperature–humidity index (THI) was calculated according to Marei et al. (2001) using the Equation (1).

(1) THI=db−[(0.31−0.31RH)(db−14.4)]

where db is the dry-bulb temperature (°C) and RH is a relative humidity (%). A value for THI < 27.8 was considered an absence of temperature stress, while a value > 28.9 was considered heat stress. In addition, the relative importance of temperature, humidity and air velocity in chicken homeostasis was investigated from a temperature–humidity–velocity index (THVI) from the Equation (2) designed by Xiuping and Xing [13].

(2) THVI=(0.85db+0.15db)−V−0.058

Equation (3) was used to calculate the homeostasis of the chickens (Tao and Xin, 2003), which calculates the variation of the body temperature (VBT) and according to it estimates the maintenance of the homeostasis when the VBT values are below 1 is considered Normal, up to 2.5 are the states of alert, between 2.5–4 are situations of danger and above 4 are states of emergency:

(3) VBT=0.39∗THVI−12.22

THI values indicated an absence of temperature stress during both periods. Values of THI were <27.8. The homeostasis value was less than 1. Homeostasis values above 1 would have been considered alarming.

### 2.4. Data Collection

The chicks were weighed when they arrived at the farm (LW0) and from then on homogeneous replicas were established which were weighed together every two weeks until slaughter (LW15 to LW120). The average daily gain (ADG) was expressed for each month (ADG30, 60, 90, 120), and the total ADG for the whole experiment (ADG 1-120), were all calculated based on the intervals between the weighing’s. The chickens were monitored daily, and to calculate mortality rates (M %), the time and date of the deaths were recorded.

Feed intake from each replicate (FI) was recorded weekly, and then calculated monthly, and as total intake (FI1-120). The feed conversion rate (FCR) was calculated on the basis of feed intake and chicken weight. The FCR was calculated for each month (FCR30, FCR60, FCR90) and for the total lifetime of the birds (FCR1-120).

To estimate homogeneity among the animals, the variability of the parameters was measured by calculating the coefficient of variation (CV) of the weekly weights.

### 2.5. Statistical Analyses

The effect of weather period and strain on weekly weight, ADG, FI, and FCR was calculated by using the general linear model procedure (GLM), with strain and weather period as a fixed effect. LW0 was used as covariance in the equation due to the difference in weights of the different breeds. Means and standard deviations were calculated for all variables. In addition, the CV between the different treatments and weeks was calculated, in order to verify the degree of uniformity per group. To test for significant differences in MR, a X2 test was used, and the Student’s t-test was calculated as a function of the period (S1 and S2) and strain (CY and RB), comparing the difference between weighing periods, and CV.

The significance level at which differences were considered was *p* < 0.05. Values 0.05 < *p* < 0.10 were considered as a trend. All statistical analyses were carried out using the SPSS Package 23 (IBM SPSS Statistic, 2017).

## 3. Results

### 3.1. Weights

Overall, at no point in the experiment did LW differ significantly between the two strains; however, in the hot period (S2), the CY chickens were significantly (*p* < 0.05) heavier at LW60, and LW75 (Table 3). No differences were found in the cold period (S2) between CY and RB.

At the LW15, chickens reared in the cold period (S1) had a significantly (*p* < 0.05) higher LW than did those reared in the warm period (Table 3), but not at LW30 and LW45. At subsequent weighings, LW was significantly (*p* < 0.05) higher in S1 than it was in S2. Thus, the difference between cold and warms periods became more pronounced as the birds aged. At the end of the experiment at 120 days, the significant differences between the groups of chickens according to the season of breeding remained, so that chickens bred in the cold period S1, weighed 383 g more than in the hot periods

### 3.2. Average Daily Gain (ADG)

In both strains and weather periods, ADG increased with age (Table 3). ADG 1–30 was significantly (*p* < 0.001) higher in the RB than it was in the CY strain, but significantly higher (*p* < 0.05) in the later at ADG 30–60. Thereafter, ADG (1–120) did not differ between the two strains. For the weather period differences (*p* < 0.05) were found in ADG 30–60, ADG 60–90, and ADG 1–120. In all cases the value was higher in the cold period (S1).

### 3.3. Feed Intake (FI)

FI was significantly (*p* < 0.05) greater in the RB than it was in the CY strain in the third month (FI 60–90), and tended to be higher in the second month (FI 30–60) (*p* = 0.08) and during the total duration of the study (FI 1–120) (*p* = 0.054) (Table 4). Feed total intake (1–120), tended to be significant (*p* = 0.054) between the two strains used, obtaining a difference of 2800 g between the RB and CY.

FI was significantly (*p* < 0.05) higher in S1 than it was in S2 in the second month (FI 30–60); however, in the third month (FI 60–90), feed intake was significantly (*p* < 0.05) higher in chickens raised in the warm period. However, no differences were found for the total duration of the study.

### 3.4. Feed Conversion Ratio (FCR)

In both strains, FCR and age were positively correlated (Table 4). In the first month (FCR 1–30), FCR was significantly (*p* < 0.01) higher in CY than in RB. In the fourth month (FCR 90–120), FCR was significantly (*p* < 0.01) higher in RB than in CY. For the total study period (1–120), the FCR did not differ significantly between strains.

The FCR did not differ significantly between the weather periods to total study time (FCR 1–120). Although, in the second month (FCR 30–60), it tended (*p* = 0.08) to be higher in the warm period than in the cold one.

### 3.5. Mortality Rates

**Figure 1:**
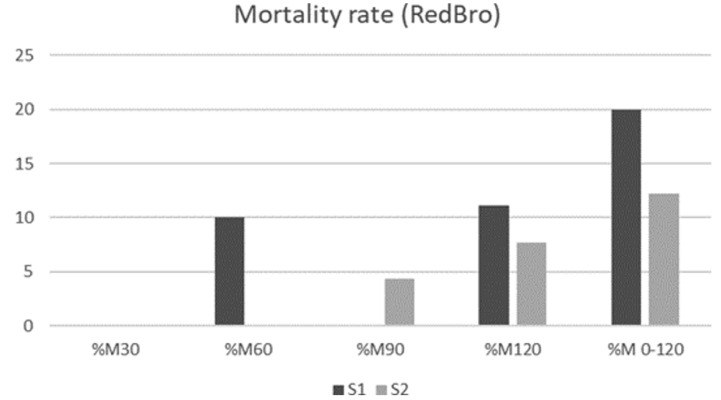
Monthly mortality rates (%M) of the RedBro strain during the two weather periods under study. %M30: Percentage of chickens died in the first month; %M60: Percentage of chickens died in the second month; %M90: Percentage of chickens died in the third month; %M90: Percentage of chickens died in the fourth month; %M120: Percentage of chickens died in the fifth month, %M0–120: Percentage of chickens died in total study period.

**Figure 2:**
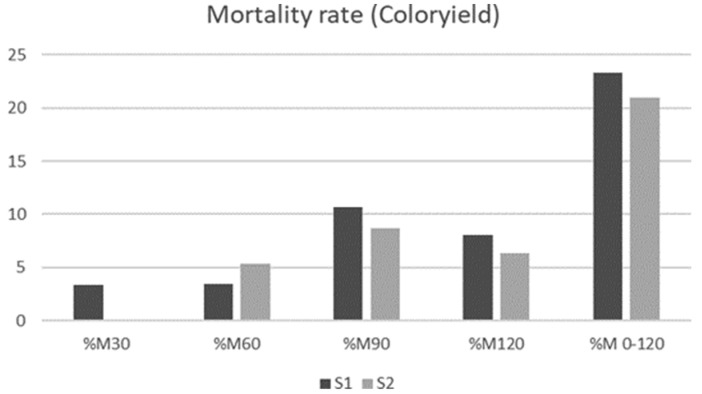
Monthly mortality rates (%M) of the Coloryield strain during the two weather periods under study. %M30: Percentage of chickens died in the first month; %M60: Percentage of chickens died in the second month; %M90: Percentage of chickens died in the third month; %M90: Percentage of chickens died in the fourth month; %M120: Percentage of chickens died in the fifth month, %M0–120: Percentage of chickens died in total study period.

Mortality rates total (M120) did not differ between strains (*p* = 0.885), or between weather periods (*p* = 0.297). The deaths started at two months of age when the animals had free access outdoors. Figure 1 and Figure 2 present mortality rates of RB and CY strains, respectively, according to weather period and life month. Relevant pathologies of dead chickens were not diagnosed.

### 3.6. Live Weight Variability

Results there were calculate at the end of rearing period (120 d of life). In both strains, LW was highly variable among individuals. Variability in LW among animals was significantly higher in the cold period than it was in the warm period at LW0 (*p* < 0.05), LW15, LW30, and LW45 (*p* < 0.01), and tended to be so at LW60, LW75, and LW90 (*p* < 0.10).

## 4. Discussion

The effects of environmental conditions on broiler performances have been extensively investigated in intensive farms, using fast-growing broilers, and under automatic ventilation and temperature control systems [14,15]. These studies have shown the influence of environmental conditions on chicken growth, FI, FCR, and survival rate. However, these studies are more limited in free-range or organic production systems, as in the present experiment, where outdoor conditions are not under control.

The THI and TVHI values shown in this study indicated the absence of stress in the chickens and normal homeostasis values for both weather periods, in accordance with what is published by Xiuping Tao et al. [13].

No differences were found between CY and RB. The LW at the end of the study for CY and RB were slightly lower than were those reported for other medium-slow growing strains [7,16]; however, the differences might have been because of differences in the ages at which the chickens were slaughtered. Castellini et al. [7] measured slaughter weight at 81 d, which is the minimum age allowed for slaughter in organic poultry farming, and [16] fixed slaughter age at 98 d. At 75 d, however, LW was higher in the CY and RB strains than it was in the slow growing strains used in those studies [16]. In any case, Bosco et al. [16] found that LW is higher in medium-growing than in pure slow-growing strains, and it is lower than fast-growing chickens. On the other hand, weather period had an effect on final LW. These differences are observed in the LW15 and disappeared in the next two weighings. From LW60 until the end of the experiment (LW120), the chickens that had been reared in S2 had a lower weight. This fact suggests the effect of climatic conditions from the first month of life of the animals, when they had access to the outdoor areas. Chickens raised under S2 had a lower weight than S1 (3.197 vs. 3.580 kg). This is in accordance with other studies [17,18,19] which showed a lower final weight at higher temperatures. It is difficult for birds to dissipate internal heat at high ambient temperatures, which leads to a decrease in food intake and a lower final weight of the chicken.

No differences were found between those lines for total ADG. Bosco et al. [16] reported results similar to this study for total ADG in medium-slow growing lines. Similar to LW, ADG was significantly higher in hybrid chickens than pure slow-growing strains. Probably, differences between the ADG in our study and those in other studies of medium-slow growing strains might have been caused the genotypes of the parents. ADG can differ between two strains of the same type of fast-growing chickens that have a different progenitor line, which suggests that the progenitor line might influence the subsequent development of the offspring and their productivity [20]. Otherwise, this study shows that ADG (1–120) was higher in chickens reared in the cold period than warm period. Similar to these results, Zhang et al. [19] showed that chickens reared at high temperatures exhibited a decrease in ADG, which became more pronounced with age. In contrast, Aksit et al. [14] showed that ADG was lowest in those chickens that had been raised at cold temperatures, and Blahová et al. [21] found that a reduction in rearing temperature did not have a significant effect on ADG.

FI was higher in RB than CY although, the reasons for these differences have not been previously described. Nor were differences found according to the rearing period for the FI. On the other hand, Blahová et al. [21] and Aksit et al. [14] reported an increase in FI in chickens raised in cold environments, and birds reared under cold stress conditions increased their feed intake [14], probably to increase and balance their body temperature. The energy of the feed is used for the maintenance of body temperature, rather than for an increase in weight, despite an increase in food intake. A reduction in intake in warm weather is an efficient mechanism for reducing excess heat and the maintenance of the homeothermy, which causes a slower growth rate [22]. However, in our study only RB showed higher FI in the cold period.

No differences were observed in the FCR depending on the line used. In both lines, FCR increased as the animals aged, which has been documented in previous studies [23]. This is caused by the increase in the FI of the chickens as they age, and because of the reduced capacity of the animals to increase their weight. The total FCR of the lines from this study, were higher than were those reported in other medium-slow growing strains [16]. Probably, the differences between studies were due to the longer duration of our experiment because, as reported elsewhere, FCR decreases as the chickens age [20,24]. In any case, the FCR of the CY and RB strains was much higher than that of fast-growing hybrids. The strains used in intensive broiler production have been selected to achieve a high weight quickly, and behave differently from strains whose selection has been less intense [25]. The high FCR of fast-growing strains is largely a consequence of having to maintain their body weight for a much shorter lifespan than is required for slow-growing strains, which have been subjected to less intense selection [16]. Although this study did not show differences in FCR for the rearing period, Blahová et al. [21] and Aksit et al. [14] reported that low temperatures had a negative effect on FCR, which has been attributed to the negative effect that cold temperature has on the immune, physiological response, and the availability of oxygen in tissues [14,21].

This study showed that chickens raised during the cold period had a higher slaughter weight (LW120), and a better total ADG (1–120). However, no differences were observed for FCR and FI. Nevertheless, RB chickens raised in cold conditions had higher LW120 (3.582 vs. 3579), FI (17.060 vs. 14.249) and FCR (5.171 vs. 4.164) than CY raised in the same period.

Feed is approximately 70% of the total cost in poultry production and, therefore, improving feed efficiency is an important objective [25]. Higher profitability is expected if FCR is low because it implies that the animal has gained more weight consuming a smaller amount of feed. In the end, the cost to the farmer is lower because less concentrate has to be purchased, and a greater economic return is derived from a higher slaughter weight. In our study, total FI in the RB strain increased slightly (*p* = 0.054), but no significant differences were detected in the other productive parameters that influence economic profitability (weight to the slaughterhouse (LW120) and FCR. Evidently, farmers can choose one line or another based on availability, without affecting economic performance. 

The difference between weather periods in animal performance can be translated into economic terms. Suppose a chicken carcass sale price of 10 currency units (c.u.)/kg. If that value is multiplied by the difference in slaughter weight (LW120) between chickens raised in the two periods (S2-S1 = −0.38 kg), the farmer will obtain a lower price (−3.80 c.u./chicken) for the animals raised in the warm period. In addition, food intake was highest in chicks raised in the warm period, although not statistically significantly so. Assuming a hypothetical cost of authorized organic feed of 0.1 c.u./kg, multiplied by the difference in per capita consumption in the two period (S1–S2 = 1.125 kg), would provide a savings in the purchase of feed for the farmer of 0.1125 c.u./chicken for the animals raised in the cold period. Clearly, chickens raised in cold-period temperatures return to the producer more economic benefit than do those raised in warm-period temperatures. Therefore, based on the productive yields and the ambient temperature only, it would be best to increase the number of animals raised in the coldest months and to reduce the number raised in the warmest months. However, farmer would need to ensure a constant supply of poultry to the market, and thus could not afford to reduce stock during warm period. So, it might be useful to study possible strategies to overcome the deleterious effects of warm conditions in organic systems.

As in the present study, Bosco et al. [16] found that mortality rates did not differ between slow- and medium-growing strains. It is likely that the absence of a significant difference in mortality between periods in this study was because temperatures below or above the thermoneutral range did not occur in our study, which produces stress on the animals, and causes physiological changes. Under those conditions, the immune system is suppressed and penetration through the intestinal lining by bacteria increases. Furthermore, thermal stress exerts negative effects on the redox balance, which exacerbates the production of reactive oxygen species [26], which contributes to the permeability of the intestinal lining to bacteria.

This study shows that CV for the LW of the two strains was high, and considerably higher than 10% reported for fast-growing chickens in intensive conventional feeding [27]. Farmers try to raise chickens that all achieve the optimum slaughter weight at the same time, which optimizes production costs [28,29]. Uniform groups obtain the most efficient overall performance, and birds reach a performance level that is close to their maximum genetic potential [30]. To meet the quality standards expected of poultry received by the processing unit, the automation used for processing carcasses at the slaughterhouse requires a uniform carcass size [31]. Events that occur in the production period strongly influence the homogeneity of the LW of the cohort. The CVs in our study were higher than were those reported for crossbreeds of autochthonous breeds at 89 d, which had CV no higher than 12.39% [32]. In fast-growing chickens, birds do not compensate for the differences in weight exhibited at hatching; therefore, farmers hope to have groups of chickens that are very homogeneous on the first day of life, which was not the case in our study. Some have suggested that chicks should be sorted into groups based on their LW at hatching once they have been received on the farm, which would reduce competition and dominance hierarchies between the largest and smallest chickens, reduce mortality, and increase homogeneity in slaughter weight [32]. Chickens raised in organic production are raised on farms that are limited to 4800 animals per unit. Farmers raise groups of 450 animals together in the same facility, which reduces competition and hierarchies. The strains that are used in organic farming are not as refined and standardized as are the strains used in intensive broiler breeding, which produce much more variation in birth weights and development.

## 5. Conclusions

Differences in the productive parameters of the two strains of slow-medium growing hybrid chickens raised in organic production systems were slight; however, ambient temperature seemed to have an impact because chickens raised in the cold season, regardless strain, reached a higher slaughter weight, although total growth rate, feed intake, and feed conversion ratio did not differ between the cold and warm seasons. Therefore, in view of these results, it might be useful to study possible strategies to overcome the deleterious effects of warm conditions in organic systems.

## Figures and Tables

**Table 1:** Ingredients and chemical composition of the diets (starter and grower-finisher).

	Starter (1–30 d)	Grower-Finisher (30–120 d)
Ingredients (g/100 g)		
Soybean meal	35.19	-
Corn	30.00	-
Wheat	12.87	30.00
Barley	9.84	30.00
Spring pea	8.00	30.00
Dicalcium phosphate	1.93	-
Calcium carbonate	0.82	-
Organic Premix ^1^	0.50	2.50
Acidifier	0.30	-
Salt	0.28	-
Sodium bicarbonate	0.16	-
Enzymatic complex	0.10	-
Sunflower	-	7.50
Chemical composition (g/kg)		
Metabolizable Energy (kcal/kg)	2462	2179
Moisture	9.24	10.16
Crude Protein	21.45	15.94
Crude Fiber	3.68	5.74
Fat	5.69	5.04
Ash	7.09	4.89

^1^ Organic Premix (Nutega Coslada, Madrid) provided the following per kilogram of diet: Calcium 1.23 g; Dry matter 4.87 g; Manganese (manganese oxide): 65.0 mg; Zinc (zinc oxide) 37.0 mg; Copper (cupric sulfate pentahydrate) 4.0 mg; Calcium iodate anhydrous: 1.90 mg; Selenium (sodium selenite) 0.10 mg; Iron (ferrous carbonate) 18.0 mg; Vitamin K3 (bisulfate menadione complex), 1.50 mg; Vitamin B2 (riboflavin) 3.00 mg; Niacinamide 15.0 mg; Calcium D-pantothenate 6.44 mg; Choline chloride 245.00 mg; Vitamin A (trans-retinyl acetate), 7500 U; Vitamin D3 (cholecalciferol) 1500 U; vitamin B12 (cyanocobalamin) 0.01 mg.

**Table 2:** Meteorological values (mean ± SD) for the two weather periods and indicators of stress according to weather conditions.

	S1	S2
MeanT (°C)	9.32 ± 0.27 ^a^	16.86 ± 0.27 ^b^
MaxT (°C)	15.57 ± 0.33 ^a^	25.06 ± 0.31 ^b^
MinT (°C)	3.61 ± 0.22 ^a^	8.96 ± 0.25 ^b^
Thermal Amplitude (°C)	11.96 ± 0.24 ^a^	16.10 ± 0.22 ^b^
Mean RH (%)	75.14 ± 0.34 ^a^	60.59 ± 0.23 ^b^
Max RH (%)	94.38 ± 0.75	87.91 ± 0.79
Min RH (%)	48.92 ± 0.33 ^a^	33.04 ± 0.34 ^b^
Wind speed (m/s)	2.05 ± 0.03 ^a^	1.55 ± 0.05 ^b^
Max Wind speed (m/s)	7.09 ± 0.08 ^a^	6.23 ± 0.13 ^b^
Solar Radiation (MJ/m^2^)	15.56 ± 0.32 ^a^	18.97 ± 0.39 ^b^
Rainfall (mm)	1.64 ± 0.11 ^a^	0.87 ± 0.18 ^b^
THI	13.77	22.65
THVI	13.21	21.72
VBT	−7.07	−3.75
Homeostasis	< 1	< 1

T: temperature; RH: relative humidity: THI: temperature–humidity index; THVI: temperature-humidity-velocity index. ^a,b^ indicate *p* < 0.0001.

**Table 3:** Mean (± SD) live weight (LW0-LW120) (kg) and monthly average daily gain (ADG 30–120) (kg/d) of two strains reared in two weather periods.

	S1 (*n* = 80)									S2 (*n* = 80)										*p*-Values	
	CY (*n* = 40)			RB (*n* = 40)			Total S1			CY (*n* = 40)			RB (*n* = 40)			Total S2			Strains	Weather Period	Interaction SxW
LW0	0.074	±	0.026	0.056	±	0.015	0.065	±	0.023	0.051	±	0.003	0.059	±	0.009	0.057	±	0.009			
LW15	0.238	±	0.076	0.237	±	0.064	0.237	±	0.070	0.188	±	0.068	0.203	±	0.035	0.200	±	0.042	0.600	0.028	0.881
LW30	0.421	±	0.147	0.484	±	0.136	0.452	±	0.144	0.407	±	0.075	0.435	±	0.069	0.430	±	0.070	0.107	0.576	0.311
LW45	0.654	±	0.168	0.728	±	0.241	0.691	±	0.209	0.680	±	0.070	0.696	±	0.120	0.693	±	0.112	0.296	0.886	0.399
LW60	1.090	±	0.295	1.253	±	0.322	1.171	±	0.316	1.040	±	0.159	0.939	±	0.217	0.957	±	0.210	0.633	0.026	0.066
LW75	1.624	±	0.421	1.824	±	0.374	1.724	±	0.407	1.499	±	0.191	1.357	±	0.263	1.383	±	0.255	0.747	0.003	0.080
LW90	2.235	±	0.455	2.307	±	0.522	2.271	±	0.486	2.035	±	0.238	1.803	±	0.336	1.845	±	0.330	0.504	0.005	0.208
LW105	2.834	±	0.506	2.905	±	0.510	2.869	±	0.504	2.638	±	0.278	2.374	±	0.395	2.422	±	0.386	0.401	0.004	0.280
LW120	3.579	±	0.667	3.582	±	0.488	3.580	±	0.578	3.108	±	0.365	3.217	±	0.466	3.197	±	0.446	0.756	0.004	0.594
ADG 1–30	0.011	±	0.004	0.014	±	0.004	0.013	±	0.004	0.011	±	0.002	0.012	±	0.002	0.012	±	0.002	0.107	0.576	0.311
ADG 30–60	0.027	±	0.007	0.020	±	0.007	0.024	±	0.008	0.026	±	0.007	0.016	±	0.006	0.023	±	0.007	0.803	0.021	0.099
ADG 60–90	0.034	±	0.007	0.038	±	0.010	0.036	±	0.008	0.030	±	0.003	0.033	±	0.010	0.031	±	0.009	0.129	0.023	0.888
ADG 90–120	0.039	±	0.018	0.042	±	0.009	0.041	±	0.014	0.031	±	0,005	0.034	±	0.015	0.033	±	0.014	0.300	0.458	0.060
ADG 1–120	0.029	±	0.005	0.029	±	0.004	0.029	±	0.004	0.025	±	0.003	0.026	±	0.003	0.026	±	0.003	0.756	0.004	0.595

ColorYield: CY, RB: RedBro, S1: cold period, S2: warm period.

**Table 4:** Mean (± SD) monthly feed intake (FI) (kg) and feed conversion ratio (FCR) of two strains of reared in the two weather periods.

	S1 (*n* = 80)			S2 (*n* = 80)			*p*-Values		
	CY (*n* = 40)	RB (*n* = 40)	Total	CY (*n* = 40)	RB (*n* = 40)	Total	Strain	Weather period	Interaction
FI 1–30	1.055 ± 0.110	1.073 ± 0.170	1.064 ± 0.143	1.111 ± 0.258	1.033 ± 0.205	1.087 ± 0.222	0.551	0.842	0.365
FI 30–60	2.523 ± 0.893	3.070 ± 1.220	2.783 ± 1.120	2.337 ± 0.334	2.567 ± 0.630	2.448 ± 0.631	0.080	0.021	0.080
FI 60–90	4.208 ± 0.256	5.519 ± 0.912	4.831 ± 0.244	5.523 ± 1.036	5.716 ± 0.244	5.593 ± 0.992	0.017	0.017	0.073
FI 90–120	6.461 ± 0.560	7.531 ± 0.985	6.969 ± 0.345	7.095 ± 0.980	6.816 ± 0.870	6.993 ± 0.860	0.127	0.874	0.011
FI 1–120	14.249 ± 6.029	17.060 ± 6.687	15.584 ± 6.296	16.770 ± 4.161	16.600 ± 3.087	16.710 ± 4.069	0.054	0.129	0.030
FCR 1–30	3.191 ± 2.316	2.488 ± 1.476	2.850 ± 1.996	5.110 ± 0.303	2.403 ± 0.565	4.126 ± 0.565	0.002	0.078	0.055
FCR 30–60	5.708 ± 1.494	5.607 ± 1.384	5.660 ± 1.443	4.465 ± 1.019	3.689 ± 2.114	4.183 ± 2.013	0.622	0.08	0.704
FCR 60–90	5.351 ± 0.989	6.513 ± 5.957	5.903 ± 4.269	5.337 ± 0.413	4.707 ± 2.087	5.108 ± 1.904	0.863	0.557	0.563
FCR 90–120	4.213 ± 2.146	6.601 ± 0.989	5.348 ± 1.659	5.357 ± 1.454	6.564 ± 1.770	5.796 ± 1.804	0.001	0.272	0.242
FCR 1–120	4.164 ± 0.631	5.171 ± 0.833	4.642 ± 0.740	4.880 ± 0.213	4.554 ± 1.302	4.767 ± 1.248	0.240	0.850	0.022

ColorYield: CY, RB: RedBro, S1: cold period, S2: warm period.
